# The Use of Metabolic Inducers in Wheat to Increase the Nutritional and Functional Value of Grain

**DOI:** 10.3390/molecules30244699

**Published:** 2025-12-08

**Authors:** Wojciech Biszczak, Izabela Jośko, Michał Świeca, Karol Kraska, Małgorzata Haliniarz, Krzysztof Różyło

**Affiliations:** 1 Department of Herbology and Plant Cultivation Techniques, University of Life Sciences in Lublin, 20-950 Lublin, Poland; 2 Institute of Plant Genetics, Breeding and Biotechnology, University of Life Sciences in Lublin, 20-950 Lublin, Poland; 3 Department of Biochemistry and Food Chemistry, University of Life Sciences in Lublin, 20-950 Lublin, Poland; 4 Faculty of Medicine, Medical University of Lublin, Al. Racławickie 1, 20-059 Lublin, Poland

**Keywords:** biostimulation, elicitors, biofortification, yield, antioxidants, amino acids, gene expression, *Acheta domesticus*, chitin

## Abstract

Stimulation of plant metabolism is a research direction for increasing the nutritional and functional value of food. In a two-year field experiment with spring wheat, eight inducers from different groups (bio- and abiotic; exo- and endogenous) were used. The tested inducers had varied and significant effects on wheat grain yield and quality. Hydrogen peroxide, chitin, and chitosan hydrochloride increased phenolic content and antioxidant activity (by 13.7%, 15.7%, and 10.1%, respectively, compared to control). Analysis of the amino acid composition of caryopses flour showed a significant increase in the content of aspartic acid, alanine, phenylalanine, and arginine after the application of hydrogen peroxide. Application of chitosan hydrochloride, L-phenylalanine, and chitin resulted in an increase in APX gene expression, while sodium hypochlorite significantly decreased CAT gene expression. Potassium iodide and sodium hypochlorite significantly reduced grain yield (by 10.6% and 14.4%, respectively, compared to control) and also worsened quality parameters of grain. Hydrogen peroxide, chitin, and chitosan hydrochloride showed the greatest stimulatory potential, as their application did not worsen, and in some cases improved, yield parameters and increased the phenolic content and antioxidant activity of grain. Hydrogen peroxide further improved the amino acid composition of grains. However, further research is needed to understand the mechanisms of effects on plants and to optimize the use of these inducers in agricultural practice.

## 1. Introduction

In addition to providing essential nutrients, food should also be a source of biologically active substances such as phenols, flavonoids, fatty acids, amino acids, and vitamins. These substances play a key role in regulating physiological processes in the bodies of consumers, including humans. Therefore, increasing their concentration in plant grains during growth has a significant impact on consumer health [1,2,3].

One of the directions of the search for environmentally neutral methods of increasing food quality and enhancing the resistance of crop plants to physical and biological stresses is the use of inducers as an agent for changing the metabolism of crop plants [4]. These methods include biofortification, which involves deliberately increasing the content of essential nutrients in foods through the use of inducers of physiological processes in plants and the production of foods with enhanced health-promoting qualities [5,6,7,8].

Inducers are substances that induce plant metabolism, activating systemic response mechanisms. They are a very important link in the regulation of processes occurring in plant cells in response to external factors. Most often, at the molecular level, they provide a signal to cells by inducing the synthesis of chemical compounds designed to nullify adverse stress factors [9,10]. The mechanisms of increasing resistance to stress factors in plants are mainly related to the production of reactive oxygen species, the “sealing” of cellular structures, the accumulation of secondary metabolites, or the synthesis of resistance-related proteins and peptides, including many defense enzymes. Stimulation with inducers takes advantage of the natural mechanisms available to plants and thus can be an environmentally neutral method of protecting crops from agrophages. Inducing stress stimuli can also significantly increase the nutritional quality of the plant food products [11,12,13]. The use of various inducers causes oxidative stress in plants, which activates a number of defense mechanisms including the expression of genes encoding antioxidant enzymes such as ascorbate peroxidase (APX), catalase (CAT), glutathione peroxidase (GPX), and superoxide dismutase (SOD), which are key to neutralizing harmful free radicals in plant cells [14,15].

Inducers used for stimulating plant metabolism can be divided according to their origin—biotic and abiotic—as well as their place of formation—endogenous and exogenous [12,16,17]. Biotic inducers are compounds both of plant origin (endogenous) and produced in the pathogen’s body (exogenous). Endogenous inducers are products of secondary metabolism of plants, e.g., nitric oxide, jasmonic acid, and salicylic acid. Exogenous ones are the building blocks of the cell walls of fungi, algae, and microorganisms, i.e., proteins, sterols, lipids, and carbohydrates, including polysaccharides (chitin and its derivatives, e.g., chitosan). Poly- and oligosaccharides have been mostly studied as signaling pathways, leading to the induction of defense responses mimicking pathogen invasion. Abiotic inducers include drought, salinity, deficiency or excess of macro and micronutrients, UV radiation, simple and complex chemicals, e.g., heavy metal salts and hydrogen peroxide, and mechanical damage [15,18].

The development of the food and feed industry based on insect farming generates waste in the form of insect chitin, which provides new opportunities for research on the issue of plant biostimulation. The cell walls of insects, crustaceans, and fungi (including pathogenic ones) are composed of chitin; hence, it is assumed that it should induce a systemic plant response [19,20,21]. Chitin is a precursor of chitosan hydrochloride, whose effectiveness as an immunity inducer in crop plants has been confirmed by numerous studies. Chitosan induces the expression of many plant genes, including those encoding glucanase and chitinase (enzymes associated with foreign pathogenic biological material). Furthermore, it increases the activity of the enzymatic antioxidant system (superoxide dismutase, catalase, and peroxidase) [19,22,23,24,25]. Based on this knowledge, it can be hypothesized that an effect comparable to that of chitosan hydrochloride can be achieved using chitin, or even an extract based on a material rich in this compound [26,27]. This is especially true given that the chitosan production process is labor-intensive and generates significant costs while producing waste in the form of chitin.

Wheat grain (*Triticum aestivum* L.) is one of the world’s most popular raw materials for feed and human food, accounting for about 30% of global grain production and 50% of global grain trade. The majority of the world’s human population considers wheat grain a basic component of their diet [28,29]. Potential biofortification with simple and inexpensive inducers under field conditions would have a large-scale effect of improving human health.

Despite promising research results on induction under controlled laboratory conditions, there is still a lack of research on the use of such inducers in modifying physiological processes of plants grown under field conditions. Therefore, the research hypothesis was to stimulate the defense mechanisms of wheat plants and, as a result, to biofortify wheat grain with the help of metabolic inducers from different groups without a significant decrease in grain yield. In turn, the aim of the study was to perform a multilevel agricultural and biochemical evaluation of the effect of using these inducers under realistic field growing conditions.

## 2. Results

### 2.1. Yield Parameters

The stimulators tested had a significant effect on spring wheat yield parameters (Table 1). Irrespective of weather factors over the years, most of the stimulators reduced grain yield compared to the control (C). Significant differences were shown for KI (potassium iodide) and SH (sodium hypochlorite). Only HP (hydrogen peroxide) and DC (deproteinized chitin) increased grain yield compared to C (control), but the differences were statistically insignificant.

The weight of crop residues was the lowest by a significant amount after the application of SH and KI relative to C. HP was the exception where trends (statistically insignificant) were observed to increase the value of this parameter. Analyzing the average of both years for the Harvest Index (HI), it was found that the value for DC was the highest, but this was not statistically significant compared to C. For thousand grain weight (TGW), there was not a statistically significant difference between the factors in both years of the experiment (Table 1). Table with standard deviations were placed in the Supplementary Materials (Table S1).

There was a significant effect of the tested inducers on spring wheat grain quality traits (Table 2). The average total protein content was significantly higher when SH was applied compared to C. Also, the application of PA (L-phenylalanine) and SA (salicylic acid) increased protein accumulation in grain compared to C, but these differences were statistically insignificant. The average gluten content of the grain was significantly higher after SH application (by 7.8% compared to C). The application of stimulants did not significantly differentiate the total starch content of wheat grains. In 2021, SH showed significantly lower total starch content compared to C. After the application of KI and SH, the Zeleny sedimentation index showed a significantly lower mean value (by 13.7% KI and by 12.9% SH) compared to C (Table 2). Tables with standard deviations were placed in the Supplementary Materials (Table S2).

### 2.2. Antioxidant Value of Grains

**Figure 1:**
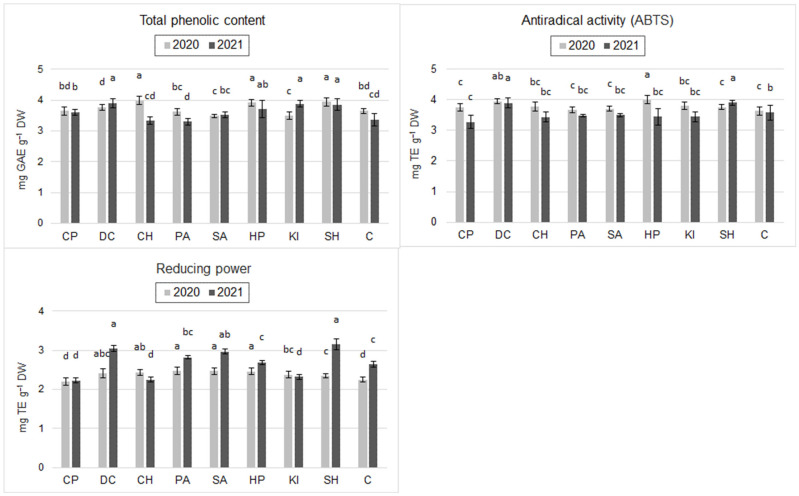
Total phenolic content (TPC), antiradical activity (ABTS) and reducing power (RP) of spring wheat grain after inducer application (mean, *n* = 6); CP—cricket powder; DC—chitin; CH—chitosan hydrochloride; PA—L-phenylalanine; SA—salicylic acid; HP—hydrogen peroxide; KI—potassium iodide; SH—sodium hypochlorite; C—control (Tukey's test; α ≤ 0.05); different letters (“abcd”) indicate statistically significant differences, while the same letters (“a”) indicate no significant differences. The bars in figures are standard deviations.

Results from 2020 showed that the application of HP, DC, and CH (chitosan hydrochloride) significantly increased the total phenolic content relative to C (by 9.3%, 9.0%, and 13.7%, respectively). The application of KI, SA, PA, and SH decreased the total phenolic content with respect to C. However, these differences were statistically insignificant (Figure 1). In 2021, the total phenolic content was significantly higher after an application of HP, DC, and CH compared to C (by 13.7%, 15.7%, and 10.1%, respectively). Also, CP, SA, and SH increased the phenolic content of the grain relative to the control, but these differences were statistically insignificant. The exception was PA, which slightly decreased phenolic content compared to C (Figure 1).

Antiradical activity (ABTS) of spring wheat grain in 2020 also showed statistically significant differences: the inducers HP, DC, and CH increased ABTS levels compared to C (by 9.9%, 8.5%, and 9.1%, respectively), while other inducers did not significantly differentiate this parameter (Figure 1). In 2021, ABTS did not change significantly after application of the inducers relative to C. However, there was a trend towards increasing values of this parameter after application of DC (8.9%) and CH (7.3%) (Figure 1).

In 2020, HP, SA, DC, and CH significantly increased reducing power (RP) by 18.2%, 9.8%, 10.7%, 22.2%, and 21.8%, respectively, compared to C. Grains treated with the other inducers had similar RP to grains from control plots. In 2021, the RP of grains was significantly higher after HP, DC, and CH induction compared to C (by 9.5%, 15.5%, and 13.6%, respectively). CP and KI reduced the value of this parameter with respect to C, but the differences were statistically insignificant (Figure 1).

### 2.3. Amino Acid Content of the Grain

**Figure 2:**
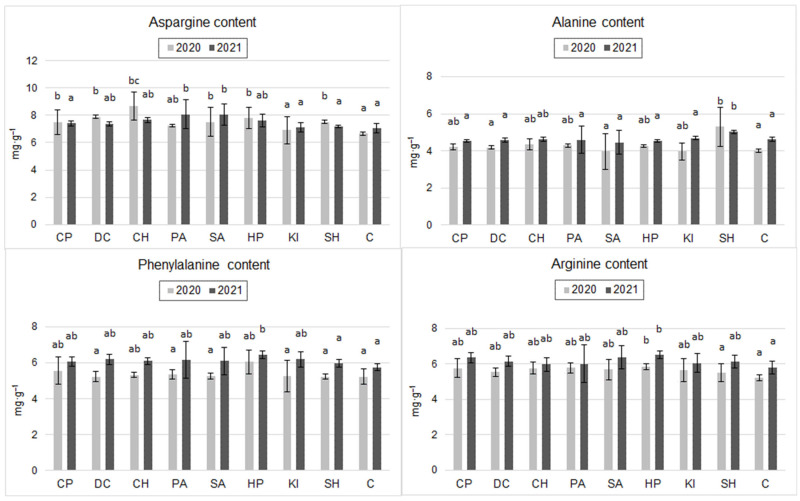
Content of selected amino acids in spring wheat grain after inducer application (mg·g^−1^) (the two years were evaluated separately; mean, *n* = 6); CP—cricket powder; DC—chitin; CH—chitosan hydrochloride; PA—L-phenylalanine; SA—salicylic acid; HP—hydrogen peroxide; KI—potassium iodide; SH—sodium hypochlorite; C—control (Tukey’s test; α ≤ 0.05); different letters (“abc”) indicate statistically significant differences, while the same letters (“a”) indicate no significant differences. The bars in figures are standard deviations.

Analysis of the amino acid composition of the grain flour (Figure 2) showed significant effects of the experimental factors only on aspartic acid (Asp), alanine (Ala), phenylalanine (Phe), and arginine (Arg). In 2020, the application of DC, SH, PA, SA, CP, HP, and CH significantly increased the Asp content of the grains with respect to C. The CH-, DC-, and HP-treated grains demonstrated the highest Asp content (30.5%, 20.2%, and 17.3% higher than in C, respectively). In 2021, differences in Arg content according to the application of the inducers tested proved statistically insignificant. Nevertheless, a clear trend of a higher content of this amino acid in the grains after the application of SA and PA was shown (Figure 2).

Ala content in 2020 was significantly highest after SH application (a 31.8% increase compared to C) (Figure 2). In 2021, SH induction increased Ala content by 9.1% compared to C, but the difference was not statistically significant (Figure 2).

In the case of the amino acid Phe, a significant increase in the content of this amino acid was observed in 2020 and 2021 after the application of HP compared to C (16% in 2020 and 12% in 2021). In 2020, the highest Arg contents in wheat grains were observed after application of HP, CP, and PA, and the values were higher than C (by 12%, 10.5%, and 10.5%), but these differences were not statistically significant. In 2021, induction with HP, CP, and SA significantly increased the Arg content of the grains (increases of 12.3%, 9.4%, and 9.3% relative to C) (Figure 2).

### 2.4. Gene Expression

Gene expression results are presented as Rq values, which are a measure of relative gene expression (RGE). A value of 1 for the control means that the gene expression levels in the control samples were taken as a baseline. The qPCR technique was used to measure gene transcript levels.

**Figure 3:**
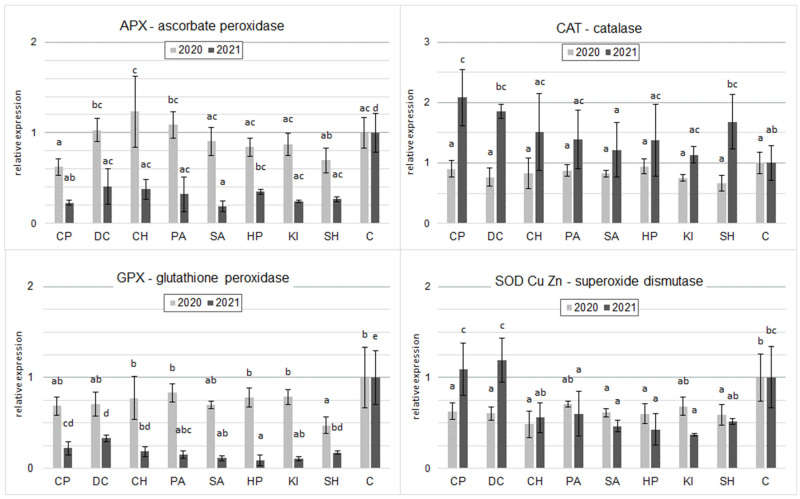
Effect of inducers on relative gene expression levels for four oxidative stress-related enzymes in spring wheat grain at milk maturity (the two years were evaluated separately; mean, *n* = 6); CP—cricket powder; DC—chitin; CH—chitosan hydrochloride; PA—L-phenylalanine; SA—salicylic acid; HP—hydrogen peroxide; KI—potassium iodide; SH—sodium hypochlorite; C—control (Tukey’s test; α ≤ 0.05); different letters (“abcde”) indicate statistically significant differences, while the same letters (“a”) indicate no significant differences. The bars in figures are standard deviations.

In the 2020 samples tested, the CH, PA, and DC inducers increased the RGE levels of APX with respect to C. However, these differences were statistically insignificant. In contrast, CP and SH significantly decreased RGE APX relative to C (Figure 3). In 2021, all inducers applied significantly reduced the level of RGE APX. The lowest level was observed after the application of SA.

In 2020, all inducers tested decreased CAT gene expression relative to controls. The differences were only significant for SH. In contrast, in 2021, all inducers increased relative CAT gene expression. A statistically proven difference was shown for CP and DC (Figure 3).

For GPX gene expression in both 2020 and 2021, all inducers reduced gene expression of this enzyme relative to the control, especially in 2021, where differences were significant for all inducers. In 2020, a statistically proven difference was shown for SH. Similar relationships were shown in the RGE of SOD Cu/Mn with the exception of CP and DC in 2021, which only tended to increase the RGE of this enzyme (Figure 3).

## 3. Discussion

Many authors have demonstrated the effects of inducers/stimulators on plant metabolism under laboratory conditions [11,12,13,15,30]. Our study demonstrated that the use of inducers that mimic biotic and abiotic stresses affects the yield, chemical composition, antioxidant properties, and relative gene expression levels of antioxidant enzymes of spring wheat grain under field growing conditions. However, these interactions are in many cases ambiguous and thus difficult to interpret.

### 3.1. The Effect of Inducers on Yield and Nutritional Value

Salicylic acid is a phenolic compound considered a plant hormone [31]. Many studies have shown that salicylic acid plays an important role in plant response to abiotic stresses [32,33]. Data collected by Abdallah et al. [34] show that treating wheat plants with different concentrations of chitosan (10 and 20 mg·L^−1^) or salicylic acid (25 and 50 mg·L^−1^) increased the carbohydrate and protein content of the grain. Using this compound, Kumar et al. [35] showed an increase in total protein content in soybean seeds. They explain this by an increase in nitrate reductase activity following the application of salicylic acid. Research by Olgun et al. [36] shows that potassium iodide is one of the better agents for simulating physiological drought stress in plants. In our study, potassium iodide significantly reduced grain yield compared to the control (by 14.2%) (Table 1). Potassium iodide also worsened most nutritional and functional quality parameters of grain (Table 2; Figure 1 and Figure 2). This means that this inducer, used for the tested dates and doses, does not fit in with the objectives of the study, i.e., grain biofortification without a significant yield decrease.

Salazar-Mercado et al. [37] showed that sodium hypochlorite at concentrations of 0.4, 1.6, and 2 mgL^−1^ inhibits *Pisum sativum* L. root apical cell division. These results differ from those reported by Causil et al. [38], who, in their test of the cytotoxic effect of sodium hypochlorite on onion root apical cells, reported no significant differences in root growth for any of the concentrations evaluated (5, 2, 1, 0.5, and 0.2 mg·L^−1^). In our study, two sprays of sodium hypochlorite (5%, 30 mL·m^−2^) had a toxic effect on wheat plants, which significantly reduced grain yield (Table 1). However, the protein and gluten content of the grain increased, but the calculated yield of protein and gluten per ha was lower than in the control (Table 2). Our study also showed a positive effect of sodium hypochlorite on the functional value of the grain, as it increased the phenolic content of the grain (Figure 1 and Figure 2).

### 3.2. The Effect of Inducers on Functional Quality

Phenols are secondary metabolites that play an important role in the plant defense system, as they have a structural function in lignification and also display antibacterial activity [39]. In our study, an increase in phenolic content was shown after treatment with hydrogen peroxide, chitin, sodium hypochlorite, and chitosan hydrochloride (Figure 1). Orzali et al. [40] showed a similar relationship using chitosan for seed treatment. The significant positive effect of chitin from crickets on phenolic content confirmed the hypothesis of our study that waste chitin as a biostimulant could be an alternative to chitosan in food biofortification. Abdallah et al. [34] found the highest increase in phenolic content in the grain of the Sakha 94 cultivar with a dose of 25 mgL^−1^ of salicylic acid and a dose of 20 mg·L^−1^ of chitosan in Gemmeiza 9. It is interesting to note that a higher dose of chitosan (20 mg·L^−1^) in Sakha 94 and a higher dose of salicylic acid (25 mg·L^−1^) in Gemmeiza 9 resulted in a significant decrease in phenolic content compared to the control. This means that the effect of different doses of chitosan and salicylic acid depends even on the cultivar and may be opposite [34].

Oxidants induce a number of biochemical reactions that are responsible for the production of compounds that protect against their toxic effects. One such mechanism is the activation of PAL (phenylalanine ammonia lyase), which can be enhanced by the production of polyphenols that affect antioxidant activity [41]. The results obtained in our study indicate that this effect occurs in wheat treated with H_2_O_2_ (HP), as a significant increase in the value of antioxidant parameters (TPC, ABTS, and RP) in the grain was recorded (Figure 1). Borges et al. [42] observed that pre-treatment of seeds and spraying of plants with H_2_O_2_ induces oxidative stress by disrupting the ROS (reactive oxygen species) homeostasis of cells and the ROS-dependent signaling network, which induces an increase in the accumulation of latent defense proteins such as ROS uptake enzymes and modulating physiological processes, thus enhancing the response to stress. Szpunar-Krok et al. [43] observed that, irrespective of the timing of treatment of the plants with 1% H_2_O_2_, after 7 days, the increase in antioxidant activity in the leaves relative to the control was significantly greater than after 1 day. The authors explain this by the fact that H_2_O_2_ initially disrupts cellular ROS homeostasis, whereas on the seventh day after the second H_2_O_2_ spray, antioxidant enzyme activity in tissues increases. Ahmad et al. [44] observed that exogenous application of H_2_O_2_ (20 and 40 mg·L^−1^) increased the length of maize shoots and roots, which was associated with higher superoxide dismutase and chlorophyll content. Using H_2_O_2_ (30% (1.0 mM), after 70 and 100 days from sowing), El-Rahman et al. [45] showed a very interesting relationship of a significant reduction (43% on average) in the natural endogenous H_2_O_2_ content of wheat flag leaves compared to the control.

The functional quality of wheat grain is largely determined by its amino acid composition [46]. It is mainly determined by environmental conditions [47,48]. In a study by Jaskiewicz and Szczepanek [49], heavy rainfall during the ripening stage of winter triticale increased the content of four amino acids: lysine, phenylalanine, histidine, and arginine. Under controlled stress conditions, plants tend to increase the amount of amino acids [50]. In our study, irrespective of the weather pattern, a positive effect of most stimulants on the content of asparagine, alanine, phenylalanine, and arginine was demonstrated (Figure 2).

### 3.3. The Effect of Inducers on the Gene Expression

Analysis of the results of the relative expression levels of antioxidant enzyme genes differed according to the inducer used, suggesting that the elicitors used in the experiment may influence plant defense against oxidative stress. The differences in the obtained expression levels of these genes between the years of the experiment may have been due to environmental factors such as the weather during the growing season (Figure 3). The main system for the removal of harmful H_2_O_2_ in plant cells under abiotic stress conditions is the glutathione–ascorbate cycle, in which the isoenzyme ascorbate peroxidase (APX) plays a key role in catalyzing the conversion of H_2_O_2_ to H_2_O [51]. APX plays an important role in the ROS inhibition system and the tolerance of plants to the stress that has occurred. H_2_O_2_ detoxification in different parts of cells is involved in AsA (ascorbic acid) homeostasis and simulates the balance in the intercellular ROS messenger network [52]. Orzali et al. [40] showed that treatment of wheat seeds with chitosan had no effect on APX activity, while our study showed an increase in APX activity, but it was not statistically significant. According to El Hadrami et al. [53], the differences between the results obtained in our study may be due to the different ways of applying this biostimulant.

CAT is a key antioxidant enzyme in plants that neutralizes harmful oxygen radicals, converting them into water and oxygen. Changes in the expression of this gene are associated with plant adaptation to stress [54,55,56]. In 2020, CAT gene expression levels were non-significantly lower after treatment with all tested inducers. In the literature, according to Gill and Tuteja [57] the decrease in CAT activity is associated with a breakdown of plant defense mechanisms following exposure to stress factors. In their studies on the application of AgNPs (silver nanoparticles) as a biostimulant, Gupta et al. [58], Shaw and Hossain [59], and Sharma et al. [60] observed an increase in CAT gene expression levels. In addition, they showed that the increase in CAT gene expression levels improved plant growth parameters. Also, in our study, this relationship was observed where an increase in CAT gene expression levels was correlated with an increase in yield after application of CP and DC in 2021 (Table 2; Figure 3).

The study by Dudziak et al. [61] showed that gene expression of individual antioxidant enzymes (CAT, APX, and GPX) in wheat plants differed in response to short-term drought and during the test. GPX gene expression after 1 h was lower than in control sprouts, but significantly and progressively increased after 3 h and 6 h and was higher compared to the control. An inverse relationship was observed for APX, in which gene expression decreased gradually over time. We can therefore surmise that the results obtained may be similar, with gene expression levels of the antioxidant enzymes tested changing over time. This indicates that in order to learn more about the effects of stress inducers on wheat and the quantity and quality of the grains it produces, the gene expression of a wider range of enzymes, the timing of sampling, and the timing of inducer application should be investigated.

In a study by Abdallah et al. [34], foliar spraying of wheat plants with chitosan (10 and 20 mg·L^−1^) or salicylic acid (25 and 50 mg·L^−1^) significantly increased the activity of antioxidant enzymes (POX, CAT, and SOD) compared to the control. The detailed data show that chitosan (10 mg L^−1^) was most effective in increasing POX and CAT activity in Sakha 94, while salicylic acid (50 mg L^−1^) was most effective in Gemmeiza 9. In contrast, in SOD, the maximum increase was obtained using salicylic acid (25 mg·L^−1^) in Sakha 94 and chitosan (20 mg·L^−1^) in Gemmeiza 9. This means that the effect of the inducers depended on the dose, the wheat variety, and the enzyme tested. Similar relationships are confirmed by our study. The authors of [34] explain this by the positive effect of chitosan on regulating the osmotic pressure of the cells, thereby increasing the uptake power of water and essential macro- and micronutrients. There is also an increase in plant organic solutes and physiological components of plant growth. In addition, there is a reduction in the accumulation of harmful free radicals and an increase in antioxidant compounds and enzyme activity. All this improves the quantity and quality of the grain yield [62].

In our study, plant material (2020 wheat grains at the milk-wax maturity stage (green grains)) showed a significant decrease in SOD Cu/Zn levels after application of all elicitors (Figure 3). Decreases in SOD enzyme levels in stressor-treated plants may be due to inactivation of the enzyme by excess ROS, inhibition of enzyme synthesis, or changes in the composition of specific sequences under stress conditions [63]. In contrast, 2021 observed a minimal, non-statistically significant increase in SOD Cu/Zn transcript levels after CP and DC application. This could be explained by the fact that mitochondria were subjected to significantly increased levels of ROS during elicitation, which may be related to the mechanism responsible for protecting mitochondria from ROS. In a genome-wide identification analysis, Jiang et al. [64] showed that SOD genes are differentially expressed in response to identical stress conditions in wheat. Changes in expression patterns may depend on the nature of the plant, the stressor levels used, and the time of exposure to the stressor [65].

## 4. Materials and Methods

### 4.1. Field Experiment

The field experiment was conducted in two growing seasons (2020 and 2021) at the Czesławice Experimental Farm (51°18′23″ N, 22°16′2″ E), belonging to the University of Life Sciences in Lublin. The test plant was spring wheat of the Goplana variety, which was grown on Haplic Luvisols soil (LVh, according to the FAO-UNESCO classification) [66], described in Table 3.

During the spring wheat growing season, total precipitation was 453.8 mm in 2020 and 489.7 mm in 2021 (Table 4). These values were significantly higher than the multi-year average (LTA) for 1991–2020. Precipitation in March 2021 was significantly lower than the LTA for that month. In March 2020, precipitation did not deviate from the LTA. The highest precipitation in 2020 was recorded in May (111.4 mm) and June (170.3 mm) and was significantly higher than the LTA. In 2021, August was the month with the highest precipitation (197.8 mm). During the spring wheat growing season (March–August), the average temperature was 1.1 °C higher in 2020 than the LTA (12.51 °C) and 0.49 °C higher in 2021. In addition, the average temperature in March was higher than the LTA, at 4.6 °C and 2.6 °C.

The field experiment included agrotechnical preparation of the field before sowing, including soil application of macronutrient fertilizers and sowing of spring wheat (450 grains per 1 m^2^). Chemical protection was limited to herbicide protection only.

The experimental factor was the application of 8 inducers:

(1) CP—micronized cricket flour extract (*Acheta domesticus*)—250 g dm·ha^−1^ (3.33 g dm·L^−1^; 300 L·ha^−1^–30 mL·m^−2^);
(2) DC—chitin isolated from micronized cricket flour—250 g dm·ha^−1^ (3.33 g dm·L^−1^; 300 L·ha^−1^–30 mL·m^−2^);
(3) CH—chitosan hydrochloride—250 g·ha^−1^ (1.33 g dm·L^−1^; 300 L·ha^−1^–30 mL·m^−2^);
(4) PA—L-phenylalanine—10 g·ha^−1^ (0.033 g·L^−1^; 300 L·ha^−1^–30 mL·m^−2^);
(5) SA—salicylic acid—41.4 g·ha^−1^ (1.0 mM; 0.138 g·L^−1^; 300 L·ha^−1^–30 mL·m^−2^);
(6) HP—hydrogen peroxide—concentration 1.5% (294 mM; 300 L·ha^−1^–30 mL·1 m^−2^);
(7) KI—potassium iodide—150 g·ha^−1^ (3 mM; 0.5%; 0.5 g·L^−1^ (0.498 g·L^−1^); 300 L·ha^−1^–30 mL·m^−2^);
(8) SH—sodium hypochlorite—5% (45 L NaClO·ha^−1^; 300 L·ha^−1^–30 mL·1 m^−2^);
(9) C—control (demineralized water without inducers).

Inducers were applied at 2 dates: at the end of tillering/beginning of stalk shooting (29–30 BBCH scale) and the milk-wax maturity of grain (77–80 BBCH scale). These are critical periods for cereals, particularly in terms of nitrogen and water requirements. This is a crucial time for fertilizer application, growth regulation, and fungicide protection. Therefore, it is also the optimal time to apply inducers and demonstrate their impact on grain quantity and quality. Inducer solutions were applied using 3-L “Marolex Master ergo 3000” (Marolex Sp.z o.o., Łomna, Poland) hand-held pressure sprayers equipped with MR1.5-90 nozzles (produced by Marolex Sp.z o.o.). Demineralized water was used as the solvent for the inducers. The control was sprayed only with demineralized water. The inductors were applied in the evening in rain-free conditions when the temperature dropped to 20 °C. The field experiment was set up in a splitblock scheme in three replications.

### 4.2. Description of Inducers

HP plays a key role in plant defense mechanisms, especially in response to environmental stresses, both biotic and abiotic. HP, which is one of the reactive oxygen species (ROS), is produced in response to oxidative stress. HP’s defensive functions in plant organisms include the regulation of a number of biological processes such as growth, cell cycle, programmed cell death, hormonal signaling, and adaptation to biotic and abiotic stresses, as documented by Gechev and Hille [67] as well as Fones and Preston [68]. In addition, HP plays an important role in regulating the expression of stress-related genes and genes encoding enzymes such as Catalase 1 (cat1) and Phenylalanine ammonia lyase (pal), making these genes useful markers of responses to ROS and specific oxidative stress signaling [69].

CP is an exogenous biotic stimulant, playing the role of a pathogen-derived stimulus. The cell wall of insects, like that of plant fungal pathogens, is composed of chitin (a derivative of chitosan), and therefore, it is assumed that it should induce a systemic response in plants [19,20]. The additional content of components of exogenous origin (proteins, peptides, lipids, and insect polysaccharides) can effectively stimulate plant growth.

SA application is often associated with positive effects on plant growth and yield under stress conditions. The beneficial effects of SA application on Rubisco and phosphoenolpyruvate carboxylase (PEPC) activity in corn plants under cadmium stress have been repeatedly observed, as they have on the net photosynthetic rate in wheat under high salinity conditions [70].

PA is a precursor of the phenylpropanoid pathway, whose products are low-molecular-weight polyphenol group compounds (lignans, lignans, and flavonoids) [71]. Foliar application of L-phenylalanine solution activates plant defense mechanisms by activating the phenylpropanoid pathway, which increases the activity of the PAL enzyme, locally initiating the synthesis of phenolic compounds. The resulting metabolites, such as ferulic acid and other derivatives, are key in protecting plants by forming protective barriers or acting as antioxidants that reduce oxidative stress damage. Foliar administration of L-phenylalanine immunizes plants against biotic threats and other stresses [72].

DC is found in insect cell walls, as it is in plant fungal pathogens. It can be used as a plant stimulant, inducing resistance outside the host organism and preparing plants for acquired systemic resistance [19,73].

CH is a basic substance used in organic crops. The effectiveness of chitosan as an inducer of crop resistance has so far been confirmed for berries, potato, cereals, or herbs [19,22]. Chitosan induces the expression of many plant genes, including genes encoding glucanase and chitinase. In addition, it increases the activity of the enzymatic antioxidant system (superoxide dismutase, catalase, and peroxidase) [23].

KI is considered a factor in abiotic stress, and applying KI to wheat leaves most accurately reflects the effects of drought [36]. Water stress triggers the formation of reactive oxygen species (ROS). Increased ROS production triggers molecular reactions producing antioxidant compounds, which are key factors in determining plant tolerance to stress, including drought [61]. Short-term drought simulation (moderate phytotoxicity) using appropriate doses and at appropriate developmental stages can also result in enriching plant cells (including grain) with biologically active substances (phenols, antioxidants, etc.), which enhance the nutritional value of the grain (biofortification).

A similar effect can be achieved using SH. SH is an inexpensive disinfectant that, at higher concentrations, chemically damages plants upon direct contact, while at lower concentrations (0.5%), it can stimulate plants to increase their metabolic activity [37]. Therefore, at appropriate dilution and application timing, it can be an interesting inducer that increases the biofunctional value of grain [74]. Additionally, products containing SH are widely recommended for their biocidal activity against plant pathogens.

The selection and dosage of inducers were determined on the basis of the available scientific literature. In addition, toxicity tests of different concentrations and doses were carried out on wheat seedlings under laboratory conditions to assess their effects on plant development. Analysis of the available data made it possible to determine the optimal doses for wheat.

### 4.3. Preparation of Cricket Powder

A freeze-drying process was used to dry frozen domestic crickets (*Acheta domesticus*), after which the material was subjected to grinding in a beater mill. In order to isolate chitin from the cricket residue, the micronized powder was subjected to degreasing and deproteinization processes. Fat removal was performed by extraction in petroleum ether, following the procedure described in AOAC 991.36 [75]. The remaining lipids were extracted from the solid using hexane (Yi et al. 2013) [76] and by applying a modified technique described by Babiker et al. [77].

Extraction of the protein fraction from fat-free cricket powder was carried out based on the method of Kim et al. [78]. For this purpose, the dry fat-free powder was dispersed in ultrapure water, and the pH of the solution was adjusted to 9.5 using 1.0 M sodium hydroxide solution while stirring for 2 h. The insoluble precipitate (waste chitin) was separated by centrifugation at 35.267× *g* for 15 min. This process was repeated three times. After drying at 40 °C, it was subjected to grinding in a ball mill and then a high-speed beater mill to obtain the maximum fineness.

### 4.4. Yield Analysis

Harvesting of whole wheat plants was carried out manually from 1 m^2^ at full maturity (92–99 BBCH scale). The obtained material was subjected to threshing with a WINTERSTEIGER LD 180 laboratory thresher (WINTERSTEIGER Seedmech GmbH, Neuengeseke, Germany). The grain was then separated from plant residues. Grain and crop residues were weighed separately, converting the yield per unit area (hectare), and the Harvest Index (HI = grain weight/(residue weight + grain weight)) was calculated. From the obtained material, samples of 300 g each were separated for further qualitative analysis.

Grain composition analysis for N, gluten, and starch content was carried out separately for each replication. The total nitrogen (TN) content of fully milled grains was determined using the Kjeldahl method, according to ISO 16634-2:2016 [79]. The total protein (TP) content was calculated based on TN, using a conversion factor of N-5.7 [80]. Wet gluten was isolated mechanically according to ISO 21415-2:2015 [81]. Starch content was determined using the Clendenning method (ICC Standard no. 122/1) [82]. The ISO 5529:2007 [83] Zeleny sedimentation test procedure consisted of suspending a prepared wheat flour sample in a lactic acid solution containing bromophenol blue, followed by shaking and a resting period, and then measuring the volume of the settled sediment to predict baking quality. The collected data were used to calibrate the grain analyzer “OmegAnalizer G”, manufactured by Bruins Instruments (Westborough, MA, USA), where automatic feeding of samples with multiple measurements allowed us to obtain representative results for the studied grain parameters.

### 4.5. Analysis of Nutritional and Health-Promoting Quality of Wheat Grain

#### 4.5.1. Sample Preparation for the Determination of Amino Acids

The analysis of amino acid composition was commissioned to an accredited laboratory (CLA UP Lublin), according to CLA/PLC/34/2019 version 3, dated 1 April 2019. The content of 15 amino acids was analyzed, but due to the need to reduce the number of results and the voluminousness of the table, the results of the study focused only on a few amino acids that were significantly affected by the factors studied, and the entire table of the content of all amino acids studied is included in the Supplementary Materials (Table S3). Acid hydrolysis of proteins to determine amino acid composition without oxidation was performed according to Davis and Thomas [84]. The ground sample was hydrolyzed at 110 °C for 20 h in 6 N HCl using an Ingos HBO 16 hydrolyzer (Prague, Czech Republic). After cooling, the solution was filtered through a G-5 funnel and evaporated on an RVO 400 vacuum evaporator. The dry residue from the vacuum flask was dissolved in 0.2 mM sodium citrate buffer, pH 2.2, and filtered through a syringe filter (hydrophilic PVDF (polyvinylidene difluoride membrane), pore sizes of 0.2 μm). The solutions were analyzed using an Ingos AAA 400 amino acid analyser equipped with a Ostion LG ANB (Prague, Czech Republic) column (0.37 × 45 cm, an average particle size of 12 µm). The separation has been performed using programmed temperature gradient (initial temperature 60 °C for early-eluting amino acids, increased to 74 °C for late-eluting fractions). Four sodium citrate buffer solutions were used for gradient elution at a flow rate of 0.35 mL·min^−1^: Buffer 1—pH 2.6, 0.2 M (0-15 min), Buffer 2—pH 3.0; 0.2 M (15-30 min), Buffer 3—pH 4.25, 0.5 M (35-55 min) and Buffer 4—pH 7.9, 1 M (55-90 min). After separation of amino acids, the column was regenerated with 0.2 N NaOH (20 min.) according to the manufacturer’s instructions. Detection was carried out using post-column derivatization with ninhydrin reagent (2% ninhydrin in methyl cellosolve with stannous chloride (SnCl_2_) as a reducing agent) at a flow rate of 0.30 mL·min^−1^. The reaction coil (250 µL volume) was maintained at 120 °C. Identification of amino acids was carried out by a photometric detector at 570 nm for all amino acids, except for proline at 440 nm [85,86,87].

#### 4.5.2. Extraction of Free and Bound Phenolic Compounds

In total, 8 mL of 0.15 mM hydrochloric acid (HCl) in 80% methanol in water (v:v) was added to 200 mg of whole-grain flour and shaken for 20 minutes at room temperature. The suspension was centrifuged (10 min, 6800× *g*), and the supernatant (free phenolic extracts) was collected. The precipitate was re-extracted twice by adding 10 mL of 80% chilled methanol. The supernatants from the three-fold extraction were combined into one final supernatant. The free phenolic extracts were concentrated using a vacuum evaporator and then supplemented with methanol to a final volume of 10 mL. After extraction of the free phenolic compounds, 20 mL of 2 M NaOH was added directly to the precipitate and shaken for 90 min at 60 °C. After alkaline hydrolysis, the solution was acidified to pH 2 with 6 M HCl and centrifuged to separate turbid precipitates. Free fatty acids and other lipid impurities in the clear solution were removed by extraction with hexane (five times). Free phenolic acids were then extracted six times with ethyl acetate. The ethyl acetate extracts were combined and evaporated to dryness, and then the bound phenolic compounds were reconstituted in 10 mL of methanol and stored at −40 °C for further use [1]. Because there were no significant differences in free phenolic content relative to bound phenolic content, the results were summed and are presented in the Results as total phenolic content.

#### 4.5.3. Contents of Phenols (TPC)

A modified Folin–Ciocalteau method [1] was used to measure total phenolic content. In total, 10 µL of H_2_O and 40 µL of diluted Folin reagent (ratio 1:5 with H_2_O) was added to 10 µL of the extract. After a 3 min incubation, 250 µL of 10% Na_2_CO_3_ solution was added. After 30 min, absorbance (wavelength of 720 nm) was measured in the samples. The total phenolic content was calculated using a calibration curve prepared for gallic acid (well-characterized phenolic compound), and the results were presented as gallic acid equivalent (GAE) per gram dry weight (DW). 

#### 4.5.4. Antiradical Activity (ABTS)

Antiradical activity was evaluated using the improved ABTS decolorization assay described by Re et al. [88]. The ABTS radical cation was obtained by reacting a 7 mM ABTS solution with 2.45 mM potassium persulfate and leaving the mixture in the dark for at least 6 h at room temperature. The ABTS solution was then diluted to an absorbance of 0.7 ± 0.05 at 734 nm using a Lambda 40 UV-Vis spectrophotometer (Perkin Elmer, Waltham, MA, USA). In total, 5 µL of extract was added to 0.2 mL of ABTS + solution, and absorbance was measured after 2 h. The ability of the material to neutralize the ABTS radical was evaluated according to the following equation:

% scavenging = ([AC − AA]/AC) × 100,

where AC is the absorbance of the control solution, and AA is the absorbance of the sample. The activity was calculated using a calibration curve prepared for Trolox (water-soluble analog of vitamin E), and the results were presented as Trolox equivalent (TE) per gram dry weight (DW)

#### 4.5.5. Reducing Power (RP)

The reducing power of the samples was determined according to the method of Oyaizu [89]. In total, 60 µL of phosphate buffer (200 mM, pH 6.6) and 60 µL of 1% potassium ferrocyanide K_3_[Fe(CN)_6_] were added to 60 µL of sample. After a 20 min incubation at 50 °C, the reaction was stopped by adding 60 µL of 10% TCA, and the samples were centrifuged for 10 min at 6500 g. In total, 100 µL of the top solution was taken, 100 µL of distilled water and 20 µL of 0.1% FeCl_3_ were added, and the absorbance was measured at 700 nm. RP results are presented as Trolox equivalent per gram of dry weight (DW).

### 4.6. Gene Expression of Antioxidant Enzymes

Isolation of total RNA and synthesis of cDNA: For gene expression studies of antioxidant enzymes, grains harvested at the milk-wax stage of development (75–80 on the BBCH scale) were used, after 24 h exposure to the stimulants under study. Immediately after the grains were separated from the ears, they were placed in Eppendorf^®^ tubes (Eppendorf, Hamburg, Germany), immediately frozen in liquid nitrogen, and later stored at −80 °C. Gene expression analyses were performed at the Institute of Plant Genetics, Breeding and Biotechnology of UP Lublin. Immediately before RNA isolation, the grains were crushed in liquid nitrogen using a mortar and pestle. To isolate total RNA from the grains, TRIzolTM reagent (Thermo Fisher Scientific Inc., Waltham, MA, USA) was used, following the procedure described by Wang et al. [90] with some modifications, as the high moisture content, abundant polysaccharides, phenolic compounds, and high endogenous RNase activity in grains probably caused RNA degradation and contamination. To optimize RNA yield and purity from seed samples, the following modifications were applied to the protocol: (I) a phenol–chloroform–isoamyl alcohol mixture (25:24:1), saturated with 100 mM TRIS solution at pH 8.0, instead of the standard phenol saturated with citrate buffer (pH 4.3) and chloroform (1:1); (II) the pH of the 3 M sodium acetate used in the process was adjusted to 5.3 instead of 4.3. The RNA isolation procedure was repeated in three independent biological experiments.

The concentration and purity of isolated RNA were measured using a NanoDrop 2000 spectrophotometer (Thermo Fisher Scientific Inc.). The integrity of the RNA samples was verified by 2% agarose gel electrophoresis, and the gel was stained with ethidium bromide. Genomic DNA was removed with DNase I (Thermo Fisher Scientific Inc.). Synthesis of cDNA from 1 µg RNA was performed using the NG dART RT kit (EURx Ltd. Gdańsk, Poland), following the manufacturer’s instructions. Subsequently, the resulting cDNA was used as a template in quantitative real-time polymerase chain reaction (qPCR) analyses.

Quantitative polymerase chain reaction analysis was performed in real time (qPCR). Expression levels of genes such as superoxide dismutase (Cu/Zn-SOD) (sequence available in GenBank under accession number U69536.1), catalase (CAT) (GenBank identifier X94352.1), ascorbate peroxidase (APX) (identifier in Phytosome Traes_2AS_007D8F7BD.1), and glutathione peroxidase (GPX) (GenBank number KP844737.1) were tested by qPCR. The primers for each gene (Table S4) were designed and optimized (the amplification rate was 90–100%). The corresponding reference genes were selected for analysis. ACT (GenBank accession number GQ339780.1) and ADP (GenBank accession number EF405961.1) were used as control genes to normalize the results.

To obtain standard curves, a dilution series was performed for each primer pair, covering five different concentrations. The qPCR procedure followed a specific thermal cycling scheme, which included initial denaturation and UNG treatment for 2 min at 50 °C, followed by denaturation for 10 min at 95 °C, and then 40 cycles, each lasting 15 s at 94 °C, 30 s at 60 °C, and 30 s at 72 °C. The total reaction volume was 25 μL and contained 1x SG/ROX qPCR Master Mix (EURx Ltd.), 500 nM of each primer, and 10 ng of cDNA. The Quant Studio3 system (Thermo Fisher Scientific) was used for qPCR analyses. Relative gene expression levels were assessed using the 2^−ΔΔCt^ method. Each sample was subjected to two technical replicates. Melting curves of PCR products were analyzed to verify specificity. To confirm the specificity of the qPCR reaction, an NTC (no template control) sample was used in each assay. Results were analyzed using the specialized Thermo Fisher Cloud software module for relative quantification, offered by Thermo Fisher Scientific.

### 4.7. Statistical Analysis

All experimental results are presented as means from three independent replicates. Statistical analysis was performed using the Statistica™ (Tibco 13.3) and “Analwar-5.FR” software. One-way analysis of variance (ANOVA, with the factor: inducer treatment) and Tukey’s post hoc test were used to compare groups with different inducers. α values less than 0.05 were considered statistically significant. Tukey’s HSD test, located between the LSD test and the Scheffé test, provides a simple method for determining the statistical significance of differences and is adequate in systems with simple factors (equal sample sizes across groups). The bars in figures are standard deviations.

## 5. Conclusions

The 2020–2021 study showed varying effects of inductors on wheat plants and partially confirmed the research hypothesis. It was observed that hydrogen peroxide and deproteinized chitin from crickets had a positive effect on grain yield parameters. The application of hydrogen peroxide, chitin, and chitosan hydrochloride increased phenolic content and antioxidant activity. Hydrogen peroxide further improved the amino acid composition of the grain. This confirms the research hypothesis of grain biofortification without a significant yield decrease for these three inducers.

Our study showed some regularities in the effect of the tested inducers but also confirmed reports from the literature that the effect of different inducers depends on many factors and can have opposite effects depending on the dose, timing of application, and other agroecological factors. Potassium iodide and sodium hypochlorite significantly reduced grain yield and also worsened most nutritional and functional quality parameters of grain. This means that these two inducers, applied at the tested dates and doses, do not fit into the objectives of the study.

Nevertheless, further research is needed to understand the mechanisms of the tested inducers’ effects on plants and to optimize their use in agricultural practice.

## Figures and Tables

**Table 1:** Yield structure of spring wheat after inducer application (mean, *n* = 3).

Trait	Year	Factor									HSD
		CP	DC	CH	PA	SA	HP	KI	SH	C	
Grain yield (t·ha^−1^)	2020	4.18 ^ab^	4.41 ^c^	4.19 ^b^	4.16 ^ab^	4.06 ^ab^	4.34 ^b^	3.75 ^a^	3.90 ^a^	4.28 ^bc^	0.24
	2021	4.13 ^b^	4.25 ^b^	3.97 ^b^	4.10 ^b^	4.08 ^b^	4.21 ^b^	3.84 ^b^	3.36 ^a^	4.19 ^b^	0.36
	Mean	4.16 ^bc^	4.29 ^c^	3.98 ^b^	4.13 ^b^	4.07 ^ab^	4.28 ^b^	3.79 ^a^	3.63 ^a^	4.24 ^bc^	0.32
Harvest residue (t·ha^−1^)	2020	6.62 ^a^	6.56 ^a^	6.55 ^a^	6.64 ^a^	6.60 ^a^	7.06 ^b^	6.59 ^a^	6.53 ^a^	6.73 ^ab^	0.21
	2021	6.44 ^b^	6.26 ^a^	6.29 ^a^	6.17 ^a^	6.31 ^a^	6.54 ^bc^	6.17 ^a^	6.05 ^a^	6.69 ^bc^	0.29
	Mean	6.53 ^ab^	6.41 ^a^	6.42 ^a^	6.41 ^a^	6.46 ^a^	6.80 ^b^	6.38 ^a^	6.29 ^a^	6.71 ^b^	0.24
Harvest Index	2020	0.39 ^a^	0.40 ^ab^	0.38 ^a^	0.39 ^a^	0.38 ^a^	0.37 ^a^	0.36 ^a^	0.37 ^a^	0.39 ^b^	0.03
	2021	0.39 ^b^	0.41 ^b^	0.39 ^b^	0.40 ^b^	0.40 ^b^	0.38 ^a^	0.38 ^a^	0.35 ^a^	0.40 ^b^	0.03
	Mean	0.39 ^ab^	0.41 ^b^	0.38 ^ab^	0.39 ^b^	0.39 ^ab^	0.38 ^a^	0.37 ^a^	0.36 ^a^	0.39 ^b^	0.03
TGW (g)	2020	30.26 ^a^	29.87 ^aa^	30.20 ^a^	30.24 ^a^	28.82 ^a^	29.9 ^ab^	29.68 ^a^	29.95 ^a^	29.48 ^a^	n.s.
	2021	30.0 ^a^	29.9 ^a^	30.0 ^a^	30.3 ^a^	29.2 ^a^	30.1 ^a^	29.8 ^a^	29.7 ^a^	29.5 ^a^	n.s.
	Mean	30.1 ^a^	29.9 ^a^	30.1 ^a^	30.3 ^a^	29.0 ^a^	30.0 ^a^	29.7 ^a^	29.8 ^a^	29.5 ^a^	n.s

CP—cricket powder; DC—chitin; CH—chitosan hydrochloride; PA—L-phenylalanine; SA—salicylic acid; HP—hydrogen peroxide; KI—potassium iodide; SH—sodium hypochlorite; C—control; “n.s.”—not significant; Harvest Index (HI = grain weight/(residue weight + grain weight)); TGW—thousand grain weight; HSD (Honestly Significant Difference (Tukey’s test; α ≤ 0.05)); different letters (“abc”) indicate statistically significant differences.

**Table 2:** Nutritional value and quality of spring wheat grain after inducer application (mean, *n* = 3).

Trait	Year	Factor									HSD
		CP	DC	CH	PA	SA	HP	KI	SH	C	
Protein content (g·kg^−1^)	2020	143.0 ^ab^	139.0 ^a^	143.0 ^ab^	145.9 ^c^	146.0 ^c^	144.3 ^bc^	140.6 ^ab^	142.1 ^ab^	142.0 ^ab^	2.8
	2021	142.1 ^a^	143.6 ^ab^	142.6 ^ab^	143.3 ^ab^	142.3 ^a^	143.6 ^ab^	140.5 ^a^	147.7 ^b^	141.07 ^a^	4.6
	Mean	142.6 ^a^	141.3 ^a^	142.8 ^a^	144.6 ^ab^	144.1 ^ab^	143.9 ^a^	140.5 ^a^	144.9 ^b^	141.5 ^a^	3.2
Wet gluten content (%)	2020	34.1 ^a^	33.5 ^ab^	34.9 ^a^	35.3 ^b^	36.1 ^bc^	33.9 ^a^	33.3 ^a^	34.0 ^a^	32.8 ^a^	2.4
	2021	35.4 ^a^	35.2 ^a^	35.2 ^a^	35.3 ^a^	35.6 ^a^	35.6 ^a^	35.3 ^a^	38.4 ^b^	34.3 ^a^	1.6
	Mean	34.7 ^ab^	34.3 ^ab^	35.0 ^ab^	35.3 ^ab^	35.7 ^ab^	34.8 ^ab^	34.3 ^ab^	36.2 ^b^	33.6 ^a^	2.0
Starch content (g·kg^−1^)	2020	524.4 ^a^	526.1 ^a^	520.9 ^a^	524.3 ^a^	521.5 ^a^	524.3 ^a^	525.9 ^a^	519.0 ^a^	520.8 ^a^	n.s.
	2021	528.3 ^b^	528.8 ^b^	529.4 ^b^	527.4 ^b^	531.8 ^b^	529.8 ^b^	530.1 ^b^	515.4 ^a^	531.0 ^b^	4.7
	Mean	526.4 ^a^	527.5 ^a^	525.2 ^a^	525.8 ^a^	526.7 ^a^	527.0 ^a^	528.0 ^a^	517.2 ^a^	525.9 ^a^	n.s
Zeleny sedimentation value	2020	35.0 ^ab^	34.6 ^ab^	34.9 ^ab^	34.8 ^ab^	36.8 ^bc^	35.8 ^b^	30.2 ^a^	31.1 ^ab^	39.0 ^c^	5.0
	2021	35.8 ^a^	37.7 ^ab^	36.8 ^a^	37.4 ^ab^	38.4 ^b^	37.7 ^ab^	36.2 ^a^	35.8 ^a^	37.8 ^b^	1.5
	Mean	35.4 ^ab^	36.2 ^b^	35.9 ^ab^	36.1 ^ab^	37.6 ^b^	36.8 ^ab^	33.2 ^a^	33.5 ^a^	38.4 ^b^	3.2

CP—cricket powder; DC—chitin; CH—chitosan hydrochloride; PA—L-phenylalanine; SA—salicylic acid; HP—hydrogen peroxide; KI—potassium iodide; SH—sodium hypochlorite; C—control; “n.s.”—not significant; HSD (Honestly Significant Difference (Tukey’s test; α ≤ 0.05)); different letters (“abc”) indicate statistically significant differences.

**Table 3:** Contents of macro- and microcomponents in the arable layer of soil.

Macroelements	Content (mg·kg^−1^)	Abundance
N-NO_3_	17.04	medium
N-NH_4_	1.05	medium
P_2_O_5_	475	very high
K_2_O	368	very high
S-SO_4_	215	very high
Mg	44	low
Microelements	Content (mg·kg^−1^)	Abundance
Mn	245.6	medium
Cu	2.9	medium
Zn	5.5	medium
Fe	1592	medium
B	1.08	low
pH in 1 M KCl	6.3	slightly acidic
C org. % d.m.	0.81	low

**Table 4:** Temperatures and precipitation patterns in 2020 and 2021.

Months	Years					
	2020		2021		LTA * 1991–2020	
	Precipitation (mm)/Temperature (°C)					
	mm	°C	mm	°C	mm	°C
March	26	4.6	14.9	2.6	37.9	2.4
April	19	8.6	58.3	6.4	42.3	8.6
May	111.4	11.2	68	11.6	70.7	13.6
June	170.3	17.4	68.3	18.6	66.8	16.9
July	67.8	18.8	82.4	22	82.2	18.9
August	59.3	20.4	197.8	17.2	54.9	18.4
Sum/Mean (September–August)	453.8.7	13.5	489.7	13	354.8	13.13

* LTA—long-term average.

## Data Availability

Data are unavailable due to privacy. The data presented in this study are available on request from the corresponding author.

## Supplementary Materials

The following supporting information can be downloaded at https://www.mdpi.com/article/10.3390/molecules30244699/s1, Table S1: Yield structure of spring wheat after inducer application (mean, *n* = 3); Table S2: Nutritional value and quality of spring wheat grain after inducer application (mean, *n* = 3); Table S3: Endo and exogenous amino acid content in spring wheat grain after inductors application (mg·g^−1^) (averages for 2020 and 2021); Table S4: The primers used in the real-time qPCR.
