# Supplementary material for: The Use of Metabolic Inducers in Wheat to Increase the Nutritional and Functional Value of Grain
Source: Molecules. 2025 Dec 8;30(24):4699. doi: 10.3390/molecules30244699 (PMC12735996; doi:10.3390/molecules30244699)
Supplement: Supplementary file 1 [file molecules-30-04699-s001.zip › molecules-3899156-supplementary.pdf]

Supplementary Materials

Table S1. Yield structure of spring wheat after inducer application (mean, n = 3).

Trait	Year	Factor									
		CP	DC	CH	PA	SA	HP	KI	SH	C	HSD
**Grain yield ($t \cdot ha^{-1}$)**	2020	4.18±0.02	4.41±0.11	4.19±0.06	4.16±0.01	4.06±0.07	4.34±0.03	3.75±0.06	3.90±0.05	4.28±0.06	0.24
	2021	4.13±0.05	4.25±0.04	3.97±0.29	4.10±0.04	4.08±0.06	4.21±0.17	3.84±0.08	3.36±0.32	4.19±0.07	0.36
	Mean	4.16±0.04	4.29±0.15	3.98±0.22	4.13±0.04	4.07±0.06	4.28±0.17	3.79±0.08	3.63±0.32	4.24±0.07	0.32
**Harvest residue ($t \cdot ha^{-1}$)**	2020	6.62±0.03	6.56±0.05	6.55±0.03	6.64±0.01	6.60±0.03	7.06±0.04	6.59±0.19	6.53±0.02	6.73±0.04	0.21
	2021	6.44±0.20	6.26±0.16	6.29±0.17	6.17±0.05	6.31±0.04	6.54±0.06	6.17±0.03	6.05±0.11	6.69±0.03	0.29
	Mean	6.53±0.16	6.41±0.20	6.42±0.18	6.41±0.26	6.46±0.16	6.8±0.29	6.38±0.26	6.29±0.27	6.71±0.04	0.24
**Harvest Index**	2020	0.387±0.002	0.402±0.008	0.377±0.026	0.388±0.007	0.381±0.004	0.370±0.002	0.363±0.006	0.374±0.003	0.389±0.002	0.03
	2021	0.396±0.005	0.410±0.006	0.391±0.019	0.396±0.008	0.396±0.009	0.381±0.010	0.382±0.011	0.354±0.012	0.400±0.010	0.03
	Mean	0.391±0.006	0.406±0.012	0.386±0.019	0.392±0.009	0.388±0.017	0.376±0.016	0.372±0.025	0.364±0.016	0.394±0.005	0.03
**TGW (g)**	2020	30.26±0.14	29.87±1.96	30.20±0.50	30.24±0.10	28.82±0.24	29.9±0.12	29.68±0.74	29.95±0.09	29.48±0.26	n.s.
	2021	30.0±0.21	29.9±0.52	30.0±0.16	30.3±0.08	29.2±0.08	30.1±0.01	29.8±0.02	29.7±0.23	29.5±0.20	n.s.
	Mean	30.1±0.21	29.9±1.14	30.1±0.38	30.3±0.25	29.0±0.57	30.0±0.11	29.7±0.50	29.8±0.34	29.5±0.29	n.s

CP—cricket powder; DC—chitin; CH—chitosan hydrochloride; PA—L-phenylalanine; SA—salicylic acid; HP—hydrogen peroxide; KI—potassium iodide; SH—sodium hypochlorite; C—control; Harvest Index (HI = grain weight / (residue weight + grain weight)); TGW—thousand grain weight; HSD (Honestly Significant Difference (Tukey's test; $\alpha \leq 0.05$)); different letters (“ab”) indicate statistically significant differences, while the same letters (“a”) indicate no significant differences.

Table S2. Nutritional value and quality of spring wheat grain after inducer application (mean, n = 3).

Trait	Year	Factor									HSD
		CP	DC	CH	PA	SA	HP	KI	SH	C	
**Protein content**	2020	143.0±1.59	139.0±1.46	143.0±0.57	145.9±0.80	146.0±0.40	144.3±0.49	140.6±0.70	142.1±0.75	142.0±0.66	2.8
**(g·kg^{-1})**	2021	142.1±2.80	143.6±0.30	142.6±1.40	143.3±2.46	142.3±1.11	143.60±2.00	140.5±0.40	147.7±1.02	141.07±0.15	4.6
	Mean	142.6±2.09	141.3±2.67	142.8±0.97	144.6±2.18	144.1±2.16	143.9±1.35	140.5±0.51	144.9±3.19	141.5±0.67	3.2
**Wet gluten**	2020	34.1±0.23	33.5±1.76	34.9±0.30	35.3±35.29	36.1±0.31	33.9±0.44	33.3±1.42	34.0±0.69	32.8±0.31	2.4
**content (%)**	2021	35.4±0.29	35.2±0.25	35.2±0.31	35.3±0.54	35.6±0.10	35.6±0.16	35.3±0.15	38.4±0.44	34.3±1.26	1.6
	Mean	34.7±0.73	34.3±1.46	35.0±0.31	35.3±0.49	35.7±0.35	34.8±0.96	34.3±1.43	36.2±2.46	33.6±1.15	2.0
**Starch content**	2020	524.4±2.15	526.1±7.07	520.9±3.96	524.3±7.20	521.5±5.62	524.3±6.37	525.9±8.43	519.0±5.72	520.8±0.86	n.s
**(g·kg^{-1})**	2021	528.3±0.64	528.8±3.15	529.4±0.96	527.4±1.76	531.8±1.60	529.8±0.38	530.1±0.50	515.4±2.51	531.0±0.70	4.7
	Mean	526.4±2.57	527.5±5.11	525.2±5.35	525.8±4.99	526.7±6.74	527.0±5.03	528.0±5.81	517.2±4.42	525.9±5.61	n.s
**Zeleny**	2020	35.0±2.22	34.6±0.91	34.9±1.72	34.8±1.99	36.8±1.25	35.8±2.62	30.2±1.55	31.1±0.59	39.0±1.63	5.0
**sedimentation value**	2021	35.8±0.58	37.7±0.52	36.8±0.49	37.4±0.24	38.4±0.80	37.7±0.34	36.2±0.12	35.8±0.16	37.8±0.64	1.5
	Mean	35.4±1.52	36.2±1.79	35.9±1.53	36.1±1.89	37.6±1.27	36.8±1.98	33.2±3.44	33.5±2.65	38.4±1.27	3.2

CP—cricket powder; DC—chitin; CH—chitosan hydrochloride; PA—L-phenylalanine; SA—salicylic acid; HP—hydrogen peroxide; KI—potassium iodide; SH—sodium hypochlorite; C—control; Harvest Index (HI = grain weight / (residue weight + grain weight)); HSD (Honestly Significant Difference (Tukey's test; $\alpha \leq 0.05$)); different letters (“ab”) indicate statistically significant differences, while the same letters (“a”) indicate no significant differences.

Table S3. Endo and exogenous amino acid content in spring wheat grain after inductors application (mg·g^{-1}) (averages for 2020 and 2021).

Aminoacid	year	Treat									HSD
		HP	CP	KI	SA	PA	DC	SH	CH	C	
Asp	2020	7.81^{b}	7.52^{b}	6.93^{a}	7.53^{b}	7.26^{ab}	7.90^{b}	7.53^{b}	8.69^{bc}	6.66^{a}	0.91
	2021	7.60^{ab}	7.43^{a}	7.13^{a}	8.04^{b}	8.09^{b}	7.39^{ab}	7.17^{a}	7.66^{ab}	7.05^{a}	0.86
	mean	7.71^{a}	7.48^{a}	7.03^{a}	7.79^{a}	7.68^{a}	7.65^{a}	7.35^{a}	8.18^{a}	6.86^{a}	
Ala	2020	4.27^{ab}	4.21^{ab}	3.97^{a}	3.98^{a}	4.31^{ab}	4.17^{a}	5.30^{b}	4.35^{ab}	4.02^{a}	1.03
	2021	4.54^{a}	4.54^{a}	4.69^{ab}	4.47^{a}	4.60^{a}	4.58^{a}	5.04^{b}	4.62^{ab}	4.62^{a}	0.42
	mean	4.41^{a}	4.38^{a}	4.33^{a}	4.23^{a}	4.46^{ab}	4.38^{a}	5.17^{b}	4.49^{ab}	4.32^{a}	0.72
Phe	2020	6.06^{b}	5.57^{ab}	5.27^{a}	5.28^{a}	5.34^{a}	5.24^{a}	5.23^{a}	5.33^{a}	5.22^{a}	0.53
	2021	6.44^{b}	6.07^{ab}	6.19^{ab}	6.11^{ab}	6.16^{ab}	6.19^{ab}	5.96^{ab}	6.10^{ab}	5.75^{a}	0.56
	mean	6.25^{b}	5.82^{ab}	5.73^{ab}	5.70^{a}	5.75^{ab}	5.72^{ab}	5.60^{a}	5.72^{ab}	5.49^{a}	0.53
Arg	2020	5.86^{a}	5.78^{a}	5.66^{a}	5.70^{a}	5.78^{a}	5.55^{a}	5.50^{a}	5.77^{a}	5.23^{a}	n.s
	2021	6.52^{b}	6.37^{b}	6.07^{a}	6.36^{ab}	6.01^{a}	6.16^{a}	6.14^{a}	5.98^{a}	5.82^{a}	0.35
	mean	6.19^{bc}	6.07^{bc}	5.86^{ab}	6.03^{b}	5.89^{ab}	5.85^{ab}	5.82^{ab}	5.87^{ab}	5.52^{a}	0.39
Thr	2020	3.38^{a}	3.20^{a}	3.07^{a}	2.96^{a}	3.29^{a}	3.11^{a}	3.03^{a}	3.19^{a}	3.09^{a}	n.s
	2021	3.85^{a}	3.65^{a}	3.79^{a}	3.59^{a}	3.73^{a}	3.75^{a}	3.70^{a}	3.76^{a}	3.81^{a}	n.s
	mean	3.62^{a}	3.43^{a}	3.43^{a}	3.28^{a}	3.51^{a}	3.43^{a}	3.37^{a}	3.48^{a}	3.45^{a}	n.s
Ser	2020	6.04^{a}	5.87^{a}	5.54^{a}	5.48^{a}	6.13^{b}	6.26^{b}	6.38^{b}	6.11^{a}	5.97^{a}	n.s
	2021	6.26^{a}	5.98^{a}	6.13^{a}	6.13^{a}	6.26^{b}	6.06^{a}	5.87^{a}	6.00^{a}	5.91^{a}	n.s
	mean	6.15^{a}	5.93^{a}	5.84^{a}	5.81^{a}	6.20^{a}	6.16^{a}	6.13^{a}	6.06^{a}	5.94^{a}	n.s
Glu	2020	42.50^{a}	42.20^{a}	39.70^{a}	40.75^{a}	41.65^{a}	40.80^{a}	43.30^{b}	42.70^{a}	38.70^{a}	n.s
	2021	42.85^{a}	42.25^{a}	41.40^{a}	43.65^{a}	42.40^{a}	43.25^{b}	41.50^{a}	42.75^{a}	41.35^{a}	n.s
	mean	42.68^{a}	42.23^{a}	40.55^{a}	42.20^{a}	42.03^{a}	42.03^{a}	42.40^{a}	42.73^{a}	40.03^{a}	n.s
Pro	2020	14.40^{a}	13.30^{a}	12.90^{a}	13.05^{a}	13.95^{a}	13.50^{a}	13.60^{a}	13.80^{a}	14.15^{a}	n.s
	2021	12.40^{a}	12.40^{a}	12.05^{a}	13.40^{a}	13.95^{a}	12.50^{a}	12.20^{a}	12.50^{a}	12.75^{a}	n.s
	mean	13.40^{a}	12.85^{a}	12.48^{a}	13.23^{a}	13.95^{a}	13.00^{a}	12.90^{a}	13.15^{a}	13.45^{a}	n.s
Gly	2020	5.20^{b}	4.98^{a}	5.04^{a}	5.06^{a}	5.17^{a}	5.00^{a}	5.23^{b}	5.22^{a}	4.81^{a}	n.s
	2021	5.33^{a}	5.22^{a}	5.32^{a}	5.75^{b}	5.87^{b}	5.28^{a}	5.21^{a}	5.27^{a}	5.06^{a}	n.s
	mean	5.27^{a}	5.10^{a}	5.18^{a}	5.41^{a}	5.52^{a}	5.14^{a}	5.22^{a}	5.25^{a}	4.94^{a}	n.s
Val	2020	4.64^{a}	4.64^{a}	4.36^{a}	4.25^{a}	4.75^{a}	4.65^{a}	4.38^{a}	5.03^{a}	4.47^{a}	n.s
	2021	5.61^{a}	5.41^{a}	5.51^{a}	5.37^{a}	5.59^{a}	5.49^{a}	5.47^{a}	5.76^{a}	5.31^{a}	n.s
mean		5.13^{a}	5.03^{a}	4.94^{a}	4.81^{a}	5.17^{a}	5.07^{a}	4.93^{a}	5.40^{a}	4.89^{a}	n.s
Tyr	2020	3.40^{a}	3.39^{a}	3.11^{a}	3.22^{a}	3.26^{a}	3.26^{a}	3.21^{a}	3.27^{a}	3.07^{a}	n.s
	2021	3.41^{a}	3.41^{a}	3.46^{a}	3.65^{a}	3.34^{a}	3.52^{a}	3.42^{a}	3.47^{a}	3.38^{a}	n.s
mean		3.41^{a}	3.40^{a}	3.29^{a}	3.44^{a}	3.30^{a}	3.39^{a}	3.32^{a}	3.37^{a}	3.23^{a}	n.s
His	2020	2.73^{a}	2.75^{a}	2.81^{a}	2.80^{a}	2.83^{a}	2.74^{a}	2.58^{a}	2.83^{a}	2.64^{a}	n.s
	2021	3.07^{a}	3.00^{a}	3.07^{a}	3.25^{a}	3.04^{a}	3.02^{a}	2.96^{a}	3.04^{a}	3.06^{a}	n.s
mean		2.90^{a}	2.87^{a}	2.77^{a}	2.94^{a}	2.85^{a}	2.95^{a}	2.92^{a}	2.91^{a}	2.75^{a}	n.s
Lys	2020	3.45^{a}	3.39^{a}	3.44^{a}	3.96^{a}	3.46^{a}	3.36^{a}	3.57^{a}	3.49^{a}	3.29^{a}	n.s
	2021	3.67^{a}	3.58^{a}	3.67^{a}	3.89^{a}	3.54^{a}	3.60^{a}	3.60^{a}	3.60^{a}	3.63^{a}	n.s
mean		6.34^{a}	6.32^{a}	6.16^{a}	6.25^{a}	6.36^{a}	6.40^{a}	6.29^{a}	6.28^{a}	6.01^{a}	n.s
Arg	2020	5.86^{a}	5.78^{a}	5.66^{a}	5.70^{a}	5.78^{a}	5.55^{a}	5.50^{a}	5.77^{a}	5.23^{a}	n.s
	2021	6.52^{b}	6.37^{b}	6.07^{a}	6.36^{ab}	6.01^{a}	6.16^{a}	6.14^{a}	5.98^{a}	5.82^{a}	0.35
mean		6.19^{bc}	6.07^{bc}	5.86^{ab}	6.03^{b}	5.89^{ab}	5.85^{ab}	5.82^{ab}	5.87^{ab}	5.52^{a}	0.39

CP – cricket powder; DC – deproteinized chitin; CH – chitosan hydrochloride; PA – L-phenyloalanine; SA – salicylic acid; HP – hydrogen peroxide; KI – potassium iodide; SH – sodium hypochlorite; C – control; HSD (Honestly Significant Difference (Tukey's test; $\alpha \leq 0.05$))

Table S4. The primers used in the real-time qPCR.

Gene	Sequences of the primer pairs (5'-3')	Gene ID
ADP	AGGTTCTTGATGCTGATGTG	EF405961.1
	CGGAGTCCAACTACTGAATG	
ACT	GGACGCACAACAGGTATC	GQ339780.1
	CGAGGTCAAGACGAAGGA	
APX	AAGATGCCACAAGGAGAG	Traes_2AS_007D8F7BD.1
	GCTCAGTGAAGTAAGAGTTG	
CAT	TCACCACAACAACCATCAT	X94352.1
	GGCAGCCAGATAGAACAC	
GPX	CCTATACAAGTTCCTGAAGTCT	KP844737.1
	GGCATAGCGGTCTACAAC	
SOD Cu-Zn	CTGGAAATGCTGGTGGAA	U69536.1
	GAGAATGGCGTCGTTACA	
